# Naphthalene Ball Ingestion Leading to Intravascular Hemolysis and Acute Kidney Injury

**DOI:** 10.7759/cureus.61291

**Published:** 2024-05-29

**Authors:** Kasireddy Sravanthi, Manojkumar G Patil, Shailaja Mane, Srinija Garlapati

**Affiliations:** 1 Pediatrics, Dr. D. Y. Patil Medical College, Hospital and Research Centre, Dr. D. Y. Patil Vidyapeeth, Pune (Deemed to be University), Pune, IND

**Keywords:** moth ball, naphthalene poisoning, hemodialysis, intravascular hemolysis, acute kidney injury

## Abstract

Naphthalene is an aromatic hydrocarbon found in mothballs, deodorizers, or insecticides. Naphthalene poisoning is not commonly seen in the pediatric age group due to its pungent odor and taste, water insolubility, and poor absorption from the gastrointestinal tract (GIT). This case report describes a five-year-old boy who experienced accidental naphthalene mothball ingestion resulting in intravascular hemolysis and acute kidney injury (AKI). Naphthalene exposure can cause severe complications, especially in children. The clinical presentation included fever, abdominal pain, vomiting, decreased urine output, and hematuria. The laboratory findings revealed hemolytic anemia, elevated serum creatinine, and proteinuria. The child received supportive treatment including intravenous fluids, packed red blood cell transfusions, and hemodialysis for AKI. Early diagnosis and intervention are crucial for a favorable outcome. This case highlights the importance of considering naphthalene poisoning in the differential diagnosis of children with hemolysis and AKI.

## Introduction

In India, naphthalene mothballs are also used as an insect repellent [1]. Routes of naphthalene exposure include dermal, ingestion, or inhalation. Naphthalene is rapidly absorbed through inhalation, and in infants, dermal absorption may occur with potential enhancement if oil has been applied beforehand [2]. Intentional exposure to naphthalene balls is uncommon. The occurrence of poisoning in children varies between 0.33% and 7.6%. Toxicity can occur either accidentally, especially in children, or intentionally in adults as a suicide attempt. The lethal dose is not known. An overdose can lead to various clinical symptoms and abnormalities in the laboratory parameters, the most prominent of which are methemoglobinemia and intravascular hemolysis [3]. Very few cases of naphthalene poisoning and its effects on children have been reported in India [4,5]. We report a case of accidental naphthalene poisoning in a child who presented with intravascular hemolysis and acute kidney injury (AKI).

## Case presentation

A five-year-old boy was referred to our center with concern for rising kidney function tests. The patient presented with a four-day history of fever, abdominal pain, and vomiting for three days. He also reported decreased urine output and had two episodes of hematuria.

Upon admission, the child was lethargic and febrile (100.3°F), with a pulse of 126 per minute, respiration rate of 50 per minute, peripheral pulses felt, saturation at 92% on room air (98% on oxygen), and blood pressure at 82/56 mmHg (less than 50th percentile). On general examination, he appeared pale, jaundiced (icteric), and had swelling in his feet (pedal edema). There were no signs of bluish discoloration (cyanosis) or enlarged lymph nodes (lymphadenopathy). The child received one bolus (rapid infusion) of isotonic saline solution. Bilateral breath sounds were diminished in the lower lobes with crackles at the bases (auscultation of the chest). On abdominal examination, the abdomen was firm, and the liver edge was palpable 2 cm below the right costal margin. He also had a mildly enlarged spleen (grade-1 splenomegaly). Further examination revealed no other significant abnormalities.

The child was treated with intravenous fluids and maximum oxygen supplementation. An emergency packed cell volume (PCV) transfusion of 10 mL/kg was performed due to his initial hemoglobin level of 3.1 g/dL.

The child remained febrile, pale, and jaundiced for the next three days, with his serum creatinine level gradually increasing, while tests for leptospirosis and malaria were negative.

**Figure 1:**
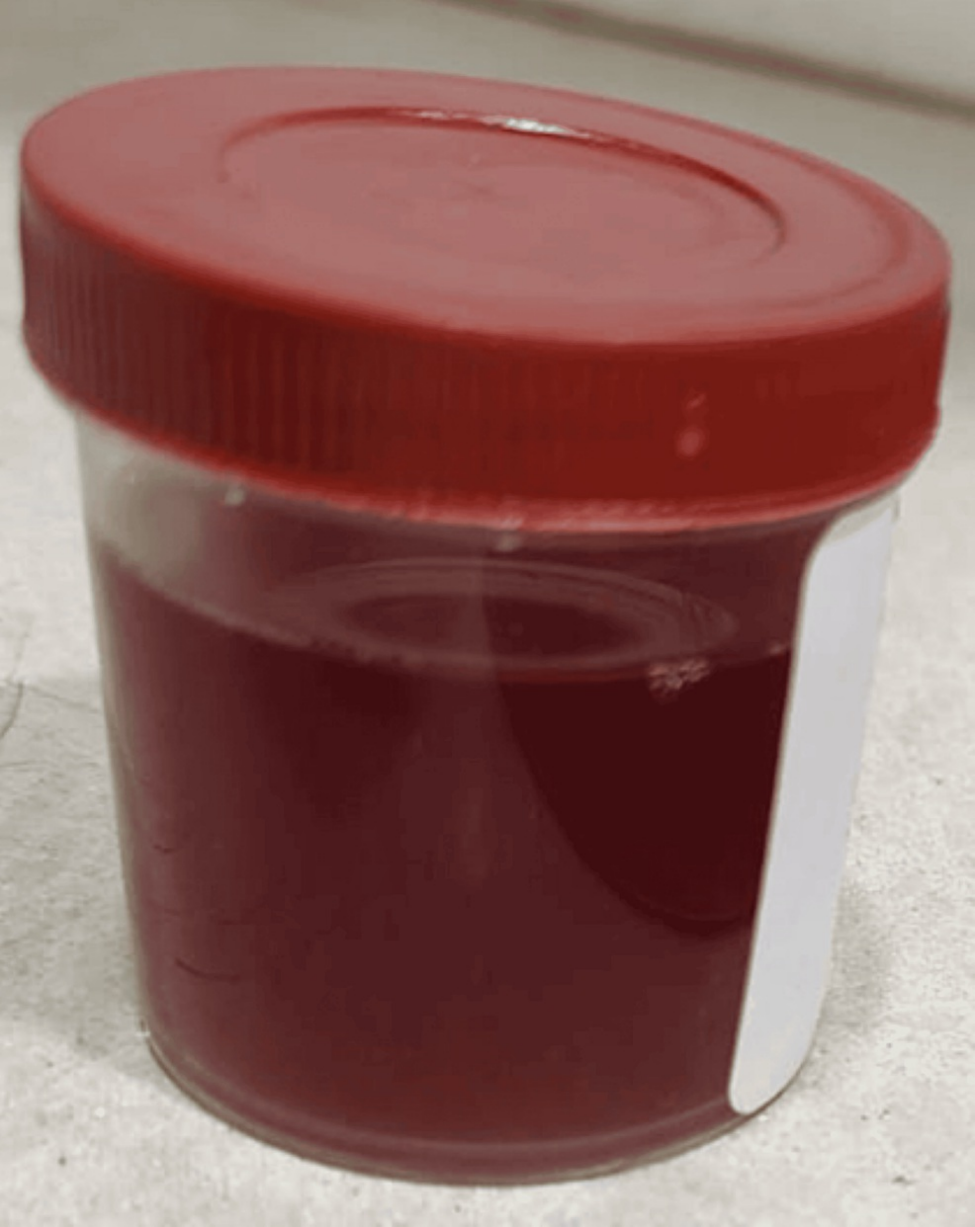
Urine sample showing characteristic red-colored appearance suggestive of hemolysis

The urine sample on visual examination was red in color as shown in Figure 1, this, along with the clinical presentation led to the suspicion of hemoglobinuria. A urine test was sent, which returned negative for hemoglobinuria, likely because the ingestion occurred four days before admission. Additionally, the hemoglobin levels were notably low at 3.1 g/dL upon presentation, suggesting significant prior hemolysis. The coagulation profile (blood clotting tests) was normal. His past medical and family history were unremarkable. Upon further questioning, the mother revealed that the child had ingested a mothball four days prior to his symptoms. Following mothball ingestion, the child developed fever, abdominal pain, and decreased urine output. With progressively worsening renal parameters, the child was started on hemodialysis. The hemodialysis was initiated with a Fresenius F4 Dialyzer machine in four intermittent sessions. The dialysate flow rate of 300 mL/min and blood flow rate of 150 mL/min were used in all four sessions, with blood flow in a countercurrent direction to dialysate flow in four sessions. Ultrafiltration volume in each session was set at 120 mL over one hour in the first session, 240 mL over two hours in the second session, 360 mL over three hours in the third session, and 500 mL over four hours in the fourth session. During each session, the patient's vital signs were closely monitored.

Further investigations, as shown in Table 1, revealed a hemoglobin level of 5.5 g/dL, a total white blood cell count of 32,000/mm³, a platelet count of 222,000/mm³, and a serum creatinine level of 5 mg/dL. A peripheral blood smear showed microcytic, hypochromic anemia with anisopoikilocytosis and schistocytes, suggestive of hemolysis. The urinalysis showed 2+ proteinuria and seven to eight red blood cells per high-power field. After four cycles, hemodialysis was discontinued as his serum creatinine was within the normal range (0.8 mg/dL). The patient received PCV transfusions to treat his anemia. The child was discharged with normal serum creatinine (0.4 mg/dL) and improved hemoglobin (11 g/dL). His blood parameters during his admission are detailed in Tables 1-3.

**Table 1:** Laboratory parameters during hospital stay SGOT: serum glutamic-oxaloacetic transaminase; SGPT: serum glutamate pyruvate transaminase; MCV: mean corpuscular volume

Investigation	Normal range	Day 1	Day 2	Day 3	Day 4	Day 6	Day 10
Hemoglobin (gm/dL)	12.0 - 14.5	3.1	3.2	5.5	8.1	7.9	9.7
Hematocrit (%)	35.7 - 43.0	8.8	9.5	15.7	23.7	23.5	30.9
Total leucocyte count (mm^3^)	4,000 - 10,800	49,000	34,000	32,000	15,700	10,000	13,800
Platelet count (mm^3^)	150,000 - 410,000	437,000	275,000	222,000	212,000	202,000	285,000
MCV (fL)	81.1 - 88	93.68	-	88.7	86.6	89.7	87.4
C-reactive protein	<3	88	-	65.5	-	45.9	-
Sodium (mmol/L)	135 - 145	132	-	132	133	137	136
Potassium (mmol/L)	3.5 - 5.0	4.5	-	4.12	3.51	3.11	3.6
Chloride (mmol/L)	98 - 110	97	-	102	98	101	98
Total bilirubin (mg/dL)	0.22 - 1.20	3.1	2	1.8	1.5	0.63	-
Direct bilirubin (mg/dL)	Upto 0.5	1	0.6	0.6	0.5	0.32	-
SGOT (U/L)	8 - 50	136	-	88	32	22	-
SGPT (U/L)	7 - 45	12	-	22	18	11	-

**Table 2:** Laboratory parameters

Investigation	Normal range	Result
Serum iron (mcg/dL)	50 - 150	306
Ferritin (ng/mL)	21.81 - 274.66	>2000
Lactate dehydrogenase (U/L)	160 - 370	2809
Reticulocyte count (%)	0.5 - 2%	7%
Sickling	-	Negative
Direct Coombs test	-	Negative
Rapid molecular test	-	Negative
Leptospira IgM/IgG	-	Negative

**Table 3:** Renal parameters

Investigation	Normal range	Day 1	Day 2	Day 3	Day 4	Day 5	Day 6	Day 10	Day 12	Day 13	Day 14
Creatinine (mg/dL)	0.19 - 0.49	2.9	4.2	5.0	6.6	5.2	2.6	3.3	2.11	1.8	0.7
Urea (mg/dL)	17 - 49	188	228	203	234	158	81	51	59	39	53

On regular follow-up, the child had normal renal, hepatic, and hematological parameters, indicating a recovery.

## Discussion

Naphthalene mothballs are commonly used in Indian households as insect repellents due to their pungent odor. It is also used as a toilet bowl deodorizer, a soil fumigant, and a component in manufacturing industrial products. It exhibits low solubility in water, and the naphthalene content in a single mothball (four grams) ranges from two grams to five grams. The occurrence of naphthalene poisoning in children varies between 0.33% and 7.6% [6]. Patients may present with complaints of vomiting and headache, diarrhea, pain in the abdomen, altered mental status, and fever. The most notable clinical sign of naphthalene poisoning is hemolysis, which results in severe kidney damage, hemoglobinuria, methemoglobinemia, anemia, and jaundice [2]. 

Naphthalene poisoning can impact various organ systems, including the nervous system, blood cells, respiratory system, and gastrointestinal tract (GIT) [1]. The degree of systemic damage depends on chemical absorption by the small intestine and the functional state of the liver. Naphthalene itself has no hemolytic property, but its oxidative metabolite, alpha-naphthol, possesses potent hemolytic properties. The toxicity of naphthalene arises from its generation of excessive oxygen free radicals, leading to lipid peroxidation and DNA damage. Naphthalene undergoes metabolism in the liver, and the resulting metabolites, such as alpha-naphthol, contribute to hemolysis and hemoglobinuria [6].

Naphthalene toxicity induces increased oxidative stress, leading to the disruption of red blood cell membranes, ultimately resulting in their destruction and intravascular hemolysis [4,7,8]. This process can contribute to AKI as intravascular hemolysis and subsequent hemoglobinuria occur [5,9,10]. The excess free hemoglobin released into the plasma overwhelms haptoglobin, an acute-phase reactant that binds to hemoglobin, forming a complex that is then cleared from the body. Consequently, the plasma levels of haptoglobin decrease. The surplus free hemoglobin, no longer bound to haptoglobin, undergoes structural changes from its usual tetrameric form to a dimer. This dimeric form can freely pass through the glomerulus and be absorbed by proximal tubular cells via the megalin-cubulin receptor system. Within these cells, hemoglobin is broken down into heme and globin [11]. The intracellular-free heme poses a threat to cellular integrity through various direct mechanisms, including lipid oxidation, protein denaturation, enzyme dysfunction, cytoskeletal instability, and DNA damage, eventually leading to acute tubular necrosis (ATN) [12-14]. Additionally, heme contributes to the formation of intratubular muddy brown pigment casts in conjunction with Tamm-Horsfall protein, while also exerting a direct cytopathic effect due to its oxidizing and pro-inflammatory properties [11]. A study by Merle et al. has highlighted the involvement of complement activation in intravascular hemolysis-induced AKI, further elucidating the complex pathophysiological mechanisms underlying this condition [15].

The treatment of naphthalene poisoning is supportive, aiming to decrease absorption and increase excretion. The management of naphthalene poisoning is symptomatic with packed red cell transfusions, fluid, and electrolyte monitoring. Hemolysis and hemoglobinuria are treated with IV hydration and packed RBCs. In managing AKI, intravenous hydration is administered to support adequate urine output. In cases of severe AKI, patients may require renal replacement therapy [2]. Naphthalene is dialyzable, making it advisable to consider consecutive dialysis treatments in cases of acute, large-volume ingestion, such as in suicide attempts, to prevent the consequences of hemolysis and ATN. Our patient had severe AKI, and hence hemodialysis was considered.

Case reports are inherently anecdotal and the findings of this case may not apply universally to all cases of naphthalene poisoning in children. However, reporting of cases like ours can offer guidance to other healthcare professionals on recognition, diagnosis, and management of similar cases in the future, while adding to the existing literature on this topic.

## Conclusions

Although Naphthalene ball poisoning is uncommon in children, it should be included among differential diagnoses of acute hemolysis with AKI. Naphthalene toxicity should be highly suspected in patients presenting with acute onset of dark brown urine, nausea, vomiting, and diarrhea, along with signs of acute hemolytic anemia, methemoglobinemia, and AKI, especially when exposure is uncertain. There are no established guidelines for treating naphthalene poisoning, which depends on the mode and severity of toxicity. Treatment primarily involves supportive care, including intravenous hydration, respiratory and blood pressure support, and potentially renal replacement therapy. Specific treatments may include ascorbic acid, methylene blue, and N-acetylcysteine (NAC). Management should be tailored to the patient's clinical severity. Early diagnosis and prompt treatment ensure a good outcome. 
